# Municipal Cross-Disciplinary Rehabilitation following Stroke in Denmark and Norway: A Qualitative Study

**DOI:** 10.1155/2018/1972190

**Published:** 2018-10-25

**Authors:** Lena Aadal, Hanne Pallesen, Cathrine Arntzen, Siri Moe

**Affiliations:** ^1^Head of Clinical Nursing Research, Cand. Cur, Ph.D., Associate Professor, Hammel Neurorehabilitation Centre and University Research Clinic, Department of Clinical Medicine, Aarhus University, Voldbyvej 15, DK-8450 Hammel, Denmark; ^2^Head of Clinical Physiotherapy Research, Cand. Scient. san., Ph.D., Associate Professor, Hammel Neurorehabilitation Centre and University Research Clinic, Department of Clinical Medicine, Aarhus University, Voldbyvej 15, DK-8450, Denmark; ^3^Associate Professor, Ph.D., Leader Centre for Care Research, North, Department of Health and Care Sciences, The Faculty of Health Sciences, UiT The Arctic University of Norway, 9037 Tromsø, Norway; ^4^Associate Professor, Ph.D., Department of Health and Care Sciences, The Faculty of Health Sciences, UiT, The Arctic University of Norway, 9037 Tromsø, Norway

## Abstract

**Aim:**

To explore and compare the content of rehabilitation practices in, respectively, a Danish and a Norwegian region, focusing on how the citizens' rehabilitation needs are met during rehabilitation in the municipalities.

**Method:**

Six Danish and five Norwegian cases were followed 12 months after the onset of stroke. Field work and focus group interviews with multidisciplinary teams in the municipalities were conducted. The conceptual frame of the International Classification of Functioning was used to outline general patterns and local variation in the rehabilitation services.

**Findings:**

Each of the settings faces different challenges and opportunities in the provision of everyday life-supportive rehabilitation services. Rehabilitation after stroke in both settings basically follows the same guidelines, but the organization of rehabilitation programmes is more specialized in Denmark than in Norway. Team organization, multidisciplinarity, and collaboration to assess and target the patients' needs characterized the Danish rehabilitation services. Decentralized coordination and monodisciplinary contributions with scarce or unsystematic collaboration were common in the Norwegian cases. Seamless holistic rehabilitation was challenged in both countries, but more notably in Norway. The municipal services emphasized physical functioning, which could conflict with the patients' needs. Cognitive disturbances to and aspects of activity or participation were systematically addressed by the interdisciplinary teams in Denmark, while practitioners in Norway found that these disturbances were scarcely addressed.

**Discussion:**

The study showed major differences in municipal stroke rehabilitation services in the Northern Norway and Central Denmark Regions—in their ability to conduct everyday life—supportive rehabilitation services. Despite the fact that biopsychosocial conceptions of disease and illness, as recommended in the ICF, have been generally accepted, they seemed scarcely implemented in the political and health managerial arenas, especially in Norway. These national diversities can partly be explained by the size of the municipalities and the available health profiles in delivering patient and family-centred rehabilitation services.

## 1. Introduction

The organization of rehabilitation interventions differs within and between European countries [1]. The establishment of acute stroke facilities and ever shorter hospital stays have led to changes in the division of rehabilitation tasks between hospitals, primary health care, and informal caregivers. In Scandinavia, the growing trend is for municipalities to take on increasing levels of responsibility [2, 3] for free public rehabilitation, with a focus on body function, activity, and participation [4]. Comparative studies have been recommended to improve the services [1]. This study describes interventions in five municipalities in Norway and two in Denmark, addressing the diversity of rehabilitation needs following stroke during patient pathways towards the re-establishment of everyday life after stroke.

## 2. Organisation of Rehabilitation

Health care in both Denmark, which has 5.7 million inhabitants, and Norway with 5.2 million comprises two complementary public sectors: the regional specialised hospital services and the primary health care system, each with separate obligations and regulated by distinctive laws and regulations. In Denmark, areas of responsibility and division of labour between regions and councils were reorganised following municipal reform in 2007 and changes in the health legislation in 2006 [5, 6]. In Norway, the Coordination Reform implemented in 2012 determined specific responsibilities between hospitals and local health authorities to develop integrated clinical pathways [7]. In both countries, the division of tasks is devolved – with the key aim of reducing in-hospital rehabilitation and widening the range of health services within the municipality. This requires major reorganization and skills transfers between the hospitals and municipal health services. Specialist health care has a statutory responsibility to provide knowledge and information to ensure the patient receives the services required after discharge. Five regions in Denmark and four in Norway are responsible for specialised hospital provision. The specialist level is distinguished in highly specialized stroke hospital units and rehabilitation at dedicated rehabilitation units. The impact of the International Classification of Function (ICF) [4], which outlines various aspects of human life that might be influenced by a stroke, has been substantial, and is increasing in the Nordic countries [8]. Both countries have clinical guidelines recommending team-based, interdisciplinary organisation in providing holistic rehabilitation, in which the timing and coordination of services are essential [2, 9]. Intermediate outreach teams have been established in both countries, to ensure seamless cross-disciplinary rehabilitation between the two governmental levels after discharge.

Once a patient is discharged from hospital, the responsibility for rehabilitation falls to the municipality. The two countries' respective primary sectors consist of 98 Danish and 428 Norwegian municipalities. Each of these provides primary health care, long-term care services, home-based care, and social care provision, accommodated to the needs of citizens and within the bounds of available economic and professional resources. The legal basis for stroke rehabilitation is regulated by a range of laws. Patients' needs generally require the participation of professionals across several fields, such as health, social care, education, and employment. Furthermore, collaboration and preclarification of the patients' rights are defined by general rules. Thus, the legal framework and sectoral legislation reflect the complexity and diversity of stroke rehabilitation [3, 10].

## 3. Diversity in Rehabilitation Frameworks in Denmark and Norway

Denmark provides centralized neurorehabilitation with a large patient volume to achieve specialization. The Danish Health Authority provides general guidelines for the organization and provision of services included in rehabilitation programs following a stroke. The Health Technology Assessment (MTV) report [10] and the Program for Progress [2] recommend that seamless rehabilitation be provided in close, coordinated, team-based, interdisciplinary collaboration, where the teams plan, prioritize, evaluate and adjust rehabilitation in close cooperation with the citizen and close relatives. The interdisciplinary team at the specialist level is required to draw up individualized rehabilitation plans upon discharge, recommending further rehabilitation provision in the municipality [11]. In determining the level of specialisation to meet the patients' needs, further specification is clarified in a clinical guideline. The overall interface of cooperation between the hospital and municipality is regulated by health agreements to ensure cross-sectorial coherence and coordination of work [2]. A majority of the Danish municipalities have engaged a brain injury coordinator to ensure a seamless, patient-oriented practice [12]. Basic-level training is often conducted by teams or therapists in public health centres and the home care staff strive to integrate rehabilitation in all everyday life activities. Other services, such as those provided by neuropsychologists, speech therapists, job consultants, social workers, or family workers, are included on an ad hoc basis by the brain injury coordinators.

The Norwegian Health Authority provides national guidelines regarding treatment and rehabilitation following a stroke [9]. Unlike Denmark, at discharge from hospital the specialist service is not not required to draw up a rehabilitation plan which commits the municipality as responsible for delivering further rehabilitation. If the patient needs further intensive rehabilitation after discharge, the general practitioner can refer the patient to a (private) rehabilitation institution with a specialist multidisciplinary treatment team [13], before the patient returns home. According to the regulations, the municipality is required to ensure the necessary professional assessment and follow-up when patients need social, psychosocial, or medical rehabilitation [13]. For patients with complex needs, the municipal responsibility encompasses the right to an individual rehabilitation plan and, since 2010, also to a personal coordinator, regardless of whether the citizen wants or asks for these initiatives.

The level of access to a contiguous coordinated intervention conducted by a specialized interdisciplinary team that works in collaboration with patients and relatives can have a major influence on rehabilitation outcome [2, 14]. Multidisciplinary rehabilitation in the patient's home environment is known to minimize dependency on care [15, 16] but there is limited knowledge about how specific therapeutic contributions in home-based rehabilitation impact functional outcome. The content of rehabilitation varies between both therapists and services [1]. The relationship between rehabilitation need and chosen therapy is unclear [17, 18], with therapists adopting an eclectic approach [19]. Furthermore, there is limited knowledge about whether the duration and content of rehabilitation programmes for stroke patients correlate with quality of services [20–22]. However, there is some knowledge about the extent to which the organization of rehabilitation after discharge from rehabilitation affects stroke patients' functioning [23]]. Since the pervasive reforms regarding the division of tasks between hospitals and municipalities, both countries have faced challenges in ensuring the efficient provision of comprehensive rehabilitation services. Innovation, reorganization, and developing innovative solutions are promoted as key aspects to meet the users' needs in the future. Therefore further knowledge about similarities and differences in patterns of the content in rehabilitation practices following stroke in a Danish and a Norwegian region, representing differences in level of specialization of treatment, population density, and geographical extent, can provide meaningful information and know how.

## 4. Aim

The aim is to describe the citizens' rehabilitation needs present at discharge after stroke and how professionals in the municipalities experience these are met, focusing on regaining a meaningful everyday life. The study is a substudy of a multicenter study “The NORDA-study”, which describes and compares stroke pathways in Norway and Denmark.

## 5. Method

Qualitative content analysis was suitable to explore and describe the multifaceted phenomena of ongoing relational rehabilitation practices [24]. As there is limited former knowledge concerning the association between the patients' rehabilitation needs and the services provided we derived categories in inductive analysis to manifest content in the texts [25]. The processing of data analysis was recursive with frequent reviews. Deductive aspects of analysis were used in the grouping of and the abstracting of categories using the conceptual framework of the ICF [4] which is a suitable tool to outline general patterns and local variations in rehabilitation practices. The ICF outlines body functions, activities and participation in daily life, taking into account environmental and personal factors, which all might be influenced by a stroke.

### 5.1. Participants

Patients: Eleven individuals, aged 25-65 and suffering from a confirmed diagnosis of stroke with moderate disability, were followed from the time of discharge from hospital until about one year after onset. The inclusion criteria were that they had lived an active, independent life before the injury and that they were discharged to their own home in one of two Danish municipalities. In Norway, we included patients consecutively, comprising their home municipalities, five in all. Professionals included were members of the municipal health services who were involved in service provision to any of the included patients. Exclusion criteria were cognitive and communication changes that made it impossible to share the patients' experiences.

### 5.2. Empirical Data

Focused field studies of each informant were used to examine interactions between patients and professionals in the municipal health services. This included conversations with professionals about their reflections, with patients about personal aims regarding and experiences of the rehabilitation process [26, 27]. Field notes were written into one complete text immediately after the observations. These data were used as a sounding board in the interpretation of the professionals' descriptions of practice and their reflections.

### 5.3. Focus Group Interviews

Ongoing rehabilitation practice from the rehabilitation professionals' perspective was explored by semi-structured focus group interviews, which allowed for exchange and the elaboration of experiences and ideas among colleagues [28–30]. The interview guide was jointly developed by all authors with main topics on the professionals' experiences, reflections, and performance, as well as user involvement of two of the included patients. Ten focus group interviews were carried out by the authors, while the professionals involved in case 11 declined participation. The interviews were audio-recorded and transcribed verbatim.

## 6. Ethical Considerations

The study was carried out according to the ethical guidelines for nursing research in the Nordic countries [31]. Permission was obtained from The Danish Data Protection Agency reference no. 1-16-02-66-14 and approved by the Regional Committees for Medical and Health Research Ethics [2013/1920]. Informed written and verbal consent to participate and access to medical records were obtained from the patients. Participation was voluntary and withdrawal was possible at any time without changes to ongoing or future rights to treatment.

## 7. Findings

### 7.1. Municipalities and Facilities

Denmark: The two Danish municipalities have 61,000 and 48,000 citizens, respectively. In each municipality, following a stroke, all citizens younger than 65 are offered rehabilitation at a health centre. The rehabilitation is organised across employment, social and health administrations as well as professional organisations in an interdisciplinary “Brainteam” affiliated to the health centre.

Norway: Patients were included from five different municipalities. Of these, four have fewer than 10,000 and the remaining municipality has 72,000 citizens. In one of the municipalities, the rehabilitation professionals involved were team organized. In the remaining municipalities, professionals worked separately as privately-practising or/and on individual locations.

### 7.2. Included Cases

See Table 1.

### 7.3. Cross-Sectoral Rehabilitation

**Figure 1:**
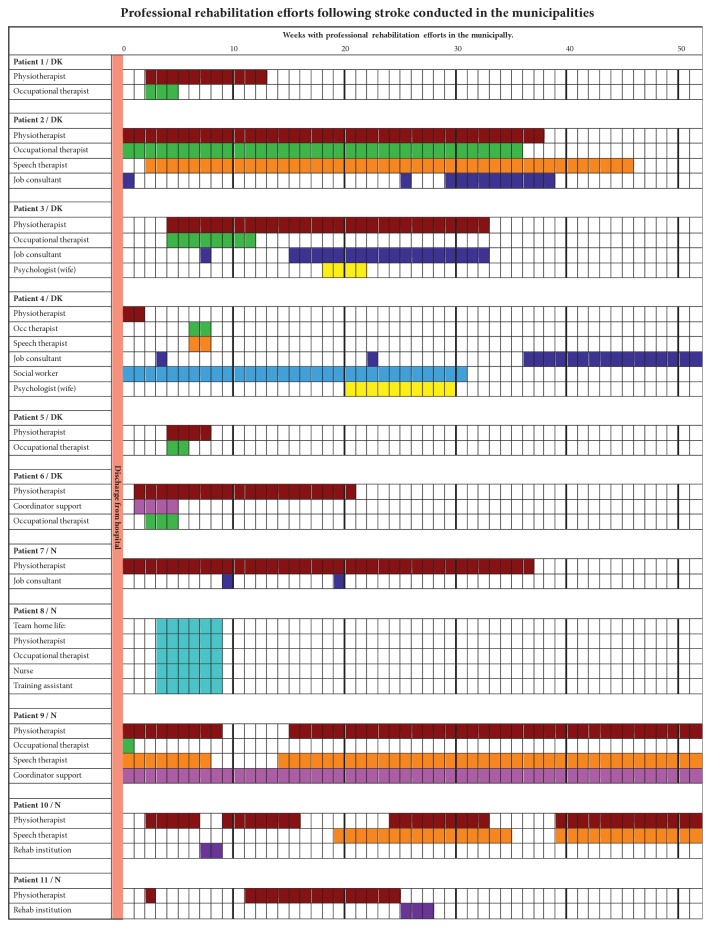
Duration of different professional rehabilitation services.

Seamless cross-sectoral rehabilitation services are hallmarks, in both Denmark and Norway. Following discharge from hospital, the municipal health services are required to facilitate coordination between different services. However, we found wide variation. In Denmark, there was a lack of continuity from discharge to municipal rehabilitation in cases 1, 3, 5, and 6. Similar discontinuity was identified in the Norwegian cases 8, 10, and 11 (Figure 1). The municipal rehabilitation was initiated with a delay of up to four weeks after discharge in Danish case 3. The explanations for the discontinuity vary between cases, but waiting lists, patients wanting a “vacation,” and communication gaps in visitation procedures were in evidence. During rehabilitation regarding the Norwegian cases 9, 10, and 11, there were weeks' long breaks in physiotherapy. The discontinuity was worsened in cases 9 and 10, as there were also gaps in speech therapist interventions caused by waiting lists and delayed applications to privately-practising therapists and rehabilitation institutions. This seemed to obstruct seamless transmission to municipal rehabilitation in Norway. One speech therapist expressed the following:

I started working with him (case 10), it was in March – where he had a referral from a doctor in [the preceding] October, but – the Helfo system [the authority who grants the treatment] rejected speech therapy  ... Only 25 hours, then a new referral from the general practitioner after a long time (case 10)

According to national recommendations in both countries, professionals in a multidisciplinary neurorehabilitation team are required to have specialist expertise in neurorehabilitation. Team members include physicians, nurses, physiotherapists (PT), occupational therapists (OT), social workers, psychologists, speech therapists, social workers, job consultants, and course coordinators. We found a wide variation in the composition of rehabilitation teams (Figure 1).

Figure 1 might show that the services were based on the patients' individual needs. Besides medical assistance, each of the included patients received physiotherapy, at the least. Occupational assessment was conducted in all Danish cases and in two of the five Norwegian cases. However there might be a gap between the initiated services and the patients' rehabilitation needs described by the team at discharge from the specialized hospital. All rehabilitation tasks were handled by the PT and speech therapist in Norwegian case 10 because all of municipal OT resources were used to administrate assistive aids. This limited specialization was also identified in other Norwegian municipalities. A PT explained that “*We do not have such a thing (neurospecialization). I'm working on rehabilitation. Everybody is working on everything” *(case 7). A privately-practicing PT questioned his own contribution: “*… it's not that I can't perform neurological physiotherapy, but I do not really see the value – as the stimulation is rare. It is only provided two or three times a week, and this intensity is not sufficient”* (case 10). In the same case, the services became monodisciplinary due to lack of coordination. Both speech therapists and physiotherapists were practicing alone, and they tried independently to support the patient's experienced needs in relation to work. However, their rehabilitation attempts seemed to fail. In describing the patient's return to work, his employer said* “… He has been thrown into normal work … supported by the other mechanics … We haven't got any instruction or supervision from professionals. We have only received a telephone call from his hospital case manager” *(case10).

Sensorimotor changes in the hand and or arm were described in all cases, except for case 2 (Table 1), but there were no OTs as fine motor rehabilitation experts between the professionals conducting rehabilitation in cases 7, 9, and 11. In case 9, cognitive disturbances were also assessed. These were detected by PT, speech therapist and coordinator, but no services were described to address this. These findings might indicate that the compositions of the rehabilitation professionals do not meet all the patients' intervention needs. The availability of professionals with the required competencies might limit the multidisciplinary services, as patients 7, 9, 10, and 11 all live in municipalities with fewer than 10,000 citizens.

### 7.4. Aspects Addressed during the Rehabilitation Process

To group and abstract the various aspects of the patient's life the professionals described to address during the rehabilitation process, the conceptual framework of the ICF was used to outline rehabilitation work related to body function, activities, and participation as well as environmental and personal factors.

**The body's functions and anatomy** refer to the physiological functions, including mental functions, and the body's anatomical structures. There appeared to be an emphasis on body functions, in that the PTs were involved in first-line work in all of the included cases and, apart from one single case (case 2), it was also the most enduring intervention. The presence of motor function as the backbone of rehabilitation was apparent, despite the absence of disturbances in this area of function. In case 4, no sensorimotor changes were described, but the patient received 38 weeks of continuous physiotherapy from discharge onwards. The initiation and duration might be explained in light of a PT assumption that improved fitness and strength improve the patient's cognitive development. The fitness training was self-organised and could therefore be understood as training in structuring: “[The training]* … stimulates him on his circuit and he is stimulated with physical activity and *[the aim is to do]* something to support the cognitive development” *(case 2), well-being “…* to exercise and doing some activity that gives her some energy” *(case 5), and participation* “… if we could keep him continuing the fitness training … he will be in a team 2-3 times a week” *(case 6), concurrently with the prevention of a new stroke.

Physiotherapy was conducted as individual self-training and as group exercises for patients with similar challenges, and the PTs and OTs emphasized the importance of time for the social aspect to facilitate recognition and participation (Table 2).

A physical focus appears in both countries, despite the prevalence of severe cognitive disturbances. Physiotherapy was offered as first-line rehabilitation, and it was also the most enduring service.

**The ICF defines activity** as the execution of a task or action by an individual [4]. The rehabilitation services were integrated into daily life activities by OTs (cases 1, 2, and 3), by the reablement team (case 8) and by the supporting pedagogue (case 4), while case 9 declined the suggested reablement. Movements associated with his work before the brain injury constituted the fulcrum of rehabilitation for the PT in case 7, while writing and counting related to work tasks were used by the speech therapist in case 10. Executions of tasks require sensorimotor as well as cognitive abilities. Motor functions were the primary OT focus in cases 1 and 8, “*some modified constrained movement therapy, where she spent fourteen days working intensively with her arm … doing daily activities with her hand at home for about 5-5.5 hours a day”* (case 1), and “*How could he hold small cherry tomatoes and the like, and the same with clothes”* (case 8).

Possible cognitive changes at admission were described in cases 1, 2, 3, 4, 6, 9, 10, and 11 (Table 1). OTs and a pedagogue described systematic individual and group-based interventions addressing realization, self-evaluation, attention, memory, planning, and accomplishing everyday tasks and how to manage energy levels. The aim of individual interventions seemed to be mainly compensatory, in that the use of a calendar, alarms, and the systematization of routines was prevalent (cases 2, 3, and 4):* “this week calendar hanging in the kitchen … she can at least sometimes see that it is actually really helpful to give her an overview of what she has not done … And, furthermore, she has some alarms on her phone* (case 4).

Professionals articulated the interaction with equals as a subsidiary goal in group-based training, to facilitate the patient's cognition and realization their condition. Therefore, both PTs and OTs were present at the training sessions to promote social interaction and to support the patients in cases 2 and 3. An OT explained that “*He started in a cognitive group … The purpose, it's really to be with someone in the same situation, and the purpose is also to gain more insight into one's own cognitive ability” *(case 2). Insight is also the explicit purpose in case 3, where the group* “shared … cognitive difficulties. In this group there has been much focus on recognition or insights”.* Case 4 was participating in an exchange of experience group, initiated by the rehabilitation team: “*A group of youths, aged about 40 years old, with dependent children living at home and something like that* [living with working spouses or partners in a consensual union]*…”.* However, interventions addressing cognitive changes were random. A Norwegian speech therapist related: “*We have worked completely systematically, where we started with clothes, colors, days, months, family members, work colleagues, and this is what we are still working on. He has a much reduced working memory and major word-finding difficulties” *(case 10). The patient is assigned 54 hours of speech therapy, but the therapist mainly focused on his severely reduced memory function without involving other health professional groups. The employer experienced a gap between the patient's needs and the rehabilitation services:* “It is very limited what he can do … with us. We try to keep him a little physically active for as long as possible. In one way, the language is not a problem for him, but it may be more to remember and such things which are more critical” *(case 10). None of the professionals mentioned the possible cognitive disturbances described at admission. The impression of inconsistent assessment and lack of provision targeted to cognitive changes were supported by the experiences of a therapist in the reablement team:* “We received specific feedback from the rehabilitation service (generalist therapists) that they can't conduct activity training … especially the occupational therapists are supposed to work with cognition. But they don't”* (case 8).

The rehabilitation services addressing activity were integrated in daily life, work, or leisure activities in the majority of both Danish and Norwegian cases. In both settings, the sensorimotor challenges appeared to be afforded a higher priority than cognitive challenges. Limited coordination between professionals resulted in fragmented and nonevidence-based rehabilitation services. This was due to monodisciplinary interventions and contributions from unsupervised employer and colleagues, which characterised the Norwegian cases. Interdisciplinary team organisation was prevalent in the Danish cases and the individualized interventions related to cognition were mainly compensatory. Group interventions and relations with equals were arranged to improve cognition and realize the changes.

**The ICF category of participation** refers to a person's involvement in everyday life situations and represents the societal perspective of functioning. Within this framework there are different roles related to the family, interpersonal interactions and relationships, employment, social life, and recreation and leisure activities. In the Danish cases, spouses and cohabitants were routinely asked to participate in planning and follow-up meetings. In addition, they might be offered training evenings about changes following a brain injury (cases 3, 5, and 6) and invited to participate in a group of relatives to exchange experiences (cases 2 and 4). Relatives' participation in rehabilitation is considered to be valuable:* “We made some clear agreements about further training, and he was so fortunate that his wife wanted to join”* (case 2). Addressing social interaction with a broader group was considered but appeared to be less prominent:* “… talked a little *(with him)* about the social … we thought about the circle of interaction. He had a family, but he seemed to be a little bit lonely” *(case 8). In case 4, the children's wellbeing was addressed; the pedagogue supported the patient to inform the children's school classes about her condition. To facilitate the patients' interaction during physical training, they were grouped in relation to the similarity of challenges and ability to benefit from each other:* “We had a really good group of gentlemen who fit really well together. Yes, they could really benefit from each other”* (case 2).

Reintegration into the social network was challenged by uncertainty and stigmatization. This was addressed in case 4, where the pedagogue described the following:* “We talked a lot about what other people think - … talked to her about informing people, in relation to her experiences of people rejecting her and that they are not reaching out”* (case 4). However, the Norwegian professionals assumed there were unmet needs:* “And in this case, relatives have got a new cohabitant/man in the family. It's a whole new person. I doubt that they take care of anything” *(case 10). In case 9, soon after discharge, the patient was offered reablement, but declined. The professionals concluded that* “She still has some trouble with anxiety. Particularly related to leaving the house … she needs as much everyday coping on the mental side” *(case 9).

Family integration was present in the Danish cases; the partners were routinely invited to planning and evaluation meetings and some of them also to training sessions. We were unable to identify any similar strategies in the Norwegian cases, but the professionals discussed the possibilities for broader social reintegration with some patients. Group training with peers, exchange of experience groups for patients and relatives, and individual support were described in the Danish settings, in order to facilitate social interaction. This aspect was not described by the Norwegian professionals.

### 7.5. Personal Aspects and Environment

According to the ICF, the personal factors of pre- and poststroke personality consist of an individual's traits that are essential to the person's behavior and ability to cope [32]. Surprisingly, we found no specific initiatives to identify and facilitate the personal cognitive factors, despite the fact that patient involvement in goal setting and daily life activities is valued:* “The prerequisite for the goals is the motivation behind training”* (case 10). Correspondingly, they acknowledge and praise when the patients are active, persistent, and responsible:* “Sh*e* has a positive energy and spreads it *… [she has]* been able to transfer the guidelines we have given her home *… [and integrate them in her activities] …* she really understands how to put training into her everyday life”* (case 1). Another highlighted a patient's approach, this time in combination with will and effort:* “*[He was]* … very optimistic … he was also very clear in the conversation with me: ‘my goal, I will return to work as a professional driver … so I exercise … You have some guts in yourself'” *(case 7).

Home visits to adapt the environment to the patients' needs were common in Denmark, but confined to one case in Norway, despite similar challenges in both groups. In all Danish cases and the Norwegian case 8, a PT and an OT visited the patients' home in order to assess needs and suggested environmental adaptation of furniture, fixtures and fittings as well as the need for aids. The need for assistive aids was also assessed by an OT in Norwegian cases 7, 8, and the 9, but there was no follow-up or involvement of rehabilitation provision. Personal aspects were acknowledged by professionals in both countries, but systematic strategies to identify and improve these were absent.

## 8. Discussion

### 8.1. Methodological Implications

This qualitative study explores and compares the rehabilitation efforts after stroke in two Danish and five Norwegian municipalities. Homogeneity in the two compared groups is not confirmed as the included participants suffered from a broad variety of changed functions. Both aspects impede the need for great caution in relation to generalize our findings to the entire national populations.

### 8.2. Seamless Rehabilitation

Rehabilitation outcome depends on access to a contiguous, coordinated intervention conducted by a specialized interdisciplinary team that collaborates with patients and relatives [10, 14]. Surprisingly, cross-sectorial continuity was challenged in this study, in that the majority of patients from municipalities in both countries experienced a time gap between one and four weeks between discharge from hospital and initiation of municipal rehabilitation. This finding is in line with the comments in reports audited by the national audit offices in Denmark and Norway [33, 34]. In Denmark, significant differences were found in waiting times between municipalities, and the Norwegian report also highlighted substantial waiting time for public as well as private initiatives. Hence, the waiting lists and lack of prioritizing might explain the prevalent fragmentation in the Norwegian rehabilitation courses, due to a delay in applications to privately-practising therapists and rehabilitation institutions, in combination with local challenges of low population density and geographical coverage in providing occupational and speech therapy [34]. Impairments did not obstruct rehabilitation at private institutions, because the included Norwegian patients managed the required levels of self-sufficiency [34]. Discontinuity might also be explained in the coordination of interventions. Both municipalities in Denmark have a brain injury coordinator; a coordination unit is lacking in four out of five of the Norwegian municipalities [34]. The general practitioner is supposed to be a key player in the coordinated unit in the Norwegian area, but this might not be the case in present practice as only case 7 had a responsibility group. The professionals in cases 9 and 10 mentioned a lack of collaboration due to missing individual rehabilitation plans or personal coordinators. Summarising our analysis of the data found that a seamless rehabilitation seemed to be inadequate in the explored settings in both countries.

### 8.3. Composition of Rehabilitation Teams

In the early weeks after a stroke, patients have varied and complex needs which require expertise from different groups of health care professionals. Both Danish municipalities have a coordinator and a specialised brain injury rehabilitation team to address various aspects of functional changes. In Norway, one patient was enrolled in reablement, while the other cases were provided generalist services in stroke rehabilitation. This entails varying competencies and resources, comparable to national findings in 2012 [33]. The variation might be explained by the differences in number of citizens, between 3,500 and 72,000 in the Norwegian municipalities involved, given that 30,000 is the recommended minimum number of citizens to ensure a qualified public service [6]. A figure below 5,000 has been found to be too low to specialise and embed specialist competencies, due to relatively few patients with specific needs [35]. A low number of patients entail fewer professionals and thus less interprofessional collaboration and organisation in multidisciplinary teams, which influences the patients' outcome [16]. Our material indicates that the Danish requirements of collaboration are embodied in the explored rehabilitation practice. One Norwegian case showed aspects of collaboration, such as rehabilitation plan, interdisciplinary meetings or shared information, vertically and horizontally (case 7), but lack of collaboration was present (cases 9 and 10). Taking the complexity of these patients' needs into consideration, the lack of a rehabilitation plan and a coordinator is surprising, given that it is required by Norwegian law; it is only known in 17% of the cases on a national basis [34]. Hence, each practitioner is expected to conduct their individual assessment of appropriate services, despite the national Norwegian obligation to consult with others. Similar lack of collaboration has been described in another study [36, 37].

### 8.4. Addressing Aspects of Life Influenced by a Stroke

National diversity was found in present health profiles, time resources and integration of rehabilitation services in the patients' everyday lives and home environment. In the Danish municipalities, the integration of rehabilitation provision in valued daily life activities was dominant, while work life movements, counting, and writing in authentic environments were more obvious in the Norwegian cases. A key emphasis on body function appears in the included cases in both settings. This indicates a discrepancy in relation to the ICF which relies on a biopsychosocial (BPS) concept of disease and illness, as formulated by Engel [38]. The main focus on physical function gives the impression of a health care system still driven by a biomedical or disease-oriented model of care. Despite the fact that BPS, as implemented in the ICF, has been generally accepted, it has been claimed that it is not used in the political and managerial areas [39] and the financial environment in the municipalities conflicts with the political aims [37]. From the professional health care worker's perspective, the ICF might appear to be merely an ideology, as it is still not integrated in rehabilitation practice.

In relation to the ICF framework of activity and participation, the patients' personal experiences and preferences related to their changed life condition should be taken into consideration [40]. Understanding the patient as collaborator in a patient-centred and individualised service implies a transformation of the patient's role from an often relegated passive, compliant recipient [41] related to the biomedical health care model. Close collaboration with the patient requires the professional core competencies of inclusion, shared decision-making, and coaching. Therefore the need for improved competencies challenges the implementation of the ICF framework as the backbone in rehabilitation services [42]. We found remarkable differences in complying the ICF recommendations, especially in relation to approaches to participation, including family roles, relationships, employment, and social life. In the Danish communities, the family members were routinely offered professional support and were included in planning processes. The patient's interaction with peers was facilitated in group training sessions or sharing of experiences. In the Norwegian cases, the professionals assumed that there were unmet needs related to participation. They addressed loneliness and issues arising from changes to personality in their conversations, but we found no specific interventions, for either the patients or their families. This may mirror professionals takes on responsibility for own trade specific and demarcated monodisciplinary contributions which challenges a holistic perspective in rehabilitation.

## 9. Conclusion

Rehabilitation after stroke basically follows the same guidelines in both settings, but the organization of rehabilitation courses is more team organized in the Danish than in the Norwegian settings. Volume and centralization seem to be pivotal in conducting rehabilitation that addresses the ICF aspects of human life influenced by a stroke. Team organization, multidisciplinarity, and collaboration to assess and target the patients' needs characterised the rehabilitation services in the two Danish municipalities. Decentralized coordination and monodisciplinary contributions with scarce or unsystematic collaboration were common in the five Norwegian municipalities. Seamless cross-sectoral services are key contributors to holistic rehabilitation. This was challenged in both countries, but most notably in Norway due to unsystematic coordination and waiting lists for privately-practising therapists. The municipal provision emphasized physical functioning in duration and intensity, which might conflict with the patients' needs, as described at discharge from hospital. Cognitive disturbances and aspects of activity or participation were systematically addressed by the interdisciplinary team in the Danish cases, while practitioners experienced a lack of multidisciplinary collaboration in the Norwegian municipalities, where these disturbances seemed to be scarcely addressed. The patterns of diversity between rehabilitation efforts in Danish and Norwegian municipalities may partly be explained by the variation in population density, geographical extent, available health profiles, time resources, and utilisation of the usual environment.

## 10. Implications

Greater consideration should be given to the aspects of activity and participation in the context of community settings. This is in order to determine how patients with stroke and those in their closest networks can be supported in becoming less dependent on public services and, as far as possible, able to understand and manage their own everyday lives. This requires an accordance between the ideology of the ICF and clinical rehabilitation practice in the political and the managerial arenas of the health care system.

## Figures and Tables

**Table 1:** Included cases. ^*∗*^In-patient days at acute stroke wards and rehabilitation hospitals/institutions.

Pt	Sex	Age	Pre injury vocation	Dwelling	Days at hospital^*∗*^	Citizes in municipality	Functional impairment at discharge from hospital (described by professionals in epicrisis, interdisciplinary status or rehabilitation plan)
1/DK	Fe	<60	pension	cohabiting	3	61.000	Sensomotor right-sided impact: Muscle strength and muscle tone slightly reduced, slightly reduced balance control. Leg lagging behind and impaired arm swing. Cognitively: Reduced executive and memory function. Fatique. Linguistic: Clearness in speech, but easy difficulty in steering the tongue.

2/DK	Ma	≤65	flexijob	cohabiting	57	61.000	Sensormotor: No functional changes. Cognitively: Reduced concentration and attention, especially persistent attention, reduced memory and cognitive language difficulties. Distinct executive difficulties with impaired idea generation, reduced work memory, lack of overview and problem solving ability. Reduced recognition and insight into own situation.

3/DK	Ma	<50	Full time	cohabiting	51	61.000	Sensomotor right sited impact: Slightly reduced strength. Reduced fitness and endurance. Cognitively: Lightly reduced memory function, lightly reduced problem solving ability, lightly troubles in word finding and left-sided lateral quadrant anopsy. Fatigue.

4/DK	Fe	<40	Full time	cohabiting and children	91	48.000	Sensomotor left sited impact: Increased tone, reduced strength as well as light changes in fine motor function in shoulder, arm and hand, increased tone in cheek and tongue. Reduced endurance. Cognitively: Difficulties in divided attention challenging to rest and maintain activity. Slightly reduced memory function and easy dysarty. Fatigue.

5/DK	Fe	<55	Part time at night	cohabiting	2	48.000	Sensomotor right sited impact: Heaviness of extremities, sensory disturbances around the mouth. Able to walk independently, but she fells a bit insecurity. Some fatigue. Cognitive. No changes

6/DK	Ma	<55	unemployed	cohabiting	37	48.000	Sensomotor left sited impact: Slight reduced control and reduced strength of the hand and leg. Left foot lags behind Balance problems, especially in the case of change of direction. Cognitive: Uncertain whether habitually or expression of cognitive change when sudden changing subject of conversation. Minor challenged in problem solving. Fatigue.

7/N	Ma	<50	Full time	cohabiting with sister and his son	28	4.800	Sensomotor right sited impact: Paralytic arm. Non-functional activation of arm and hand, but can easily activate both flexors and extensors in arm as well as supination and pronation. Incipient hand grip and dorsal reflection by hand. Reduced strength 3-4 / 5 in leg but walks without support indoors. Slight facial paresis. Cognitive: No disturbances.

8/N	Ma	≤65	Partial pension	Single	37	72.000	Sensomotor right sited impact: Reduced strength in the leg, but is able to walk with support, Reduced strength and fine motor movement in hand and fingers but has an important support function. Dysarthria. No cognitive disturbances

9/N	Fe	<50	pension	single	57	9.500	Sensomotor: Slightly tense right arm. Going independently indoor and with surveillance also outdoor. Dizziness, double vision. Cognitive: Reduced memory. Slight word finding difficulty, not fluent speech. Easily distracted by noise, other people and mess, but manage to move on in activity. Easily tired and need small breaks

10/N	Ma	<45	Full time	Cohabiting and children	56 +14 in a later rehabilitation course	3.500	Sensomotor right sited impact: Reduced stability in hip, knee, reduced quadriceps activation. Reduced strength and stability in shoulder and elbow. Walks short distances in-door. Reduced fine motor precision in hand. Cognitive: Suspected but not assessed due to language problems. Has aphasia, word finding challenges. Reduced memory. Possibly neglect in right site of his environment, still double vision forward and toward left.

11/N	Ma	<60	Partial pension	Cohabiting and child	29	5.500	Sensomotor left sited impact: Reduced sensitivity in hand/arm. Reduced attention to and stability in shoulder. Unimpressive walk without fall risk No evidence of cognitive difficulties in daily activities. Identified reduced attention to the left and in the center of view.

**Table 2:** Physiotherapeutic contributions in the eleven cases during rehabilitation.

Case	Individual 1:1 start number per week	Self training in centre primary focus on strength, condition with PT instruction number per week	Group exercise in centre with selected peers instructed by OT/PT	Public fitness centre without instruction
1/DK	2	1	1	
2/DK		3	1	
3/DK		1	1	
4/DK	1			1-2
5/DK	1	1	1	1
6/DK	3	2	2	
7/N		3		
8/N	5			
9/N	2			
10/N		3-2		
11/N	2		5	

## Data Availability

The empirical data used to support the findings of this study are included within the article.
